# Oral versus intravenous proton pump inhibitors in preventing re-bleeding for patients with peptic ulcer bleeding after successful endoscopic therapy

**DOI:** 10.1186/1471-230X-12-66

**Published:** 2012-06-08

**Authors:** Hsu-Heng Yen, Chia-Wei Yang, Wei-Wen Su, Maw-Soan Soon, Shun-Sheng Wu, Hwai-Jeng Lin

**Affiliations:** 1 Department of Gastroenterology, Changhua Christian Hospital, Changhua, Taiwan; 2 Division of Gastroenterology and Hepatology, Department of Internal Medicine, Taipei Medical University Hospital, Taipei Medical University, No. 252, Wuxing St, Taipei 11031, Taiwan

**Keywords:** Peptic ulcer bleeding, Proton pump inhibitor, Endoscopic therapy, Hemostasis, Peptic ulcer, High risk

## Abstract

**Background:**

High dose intravenous proton pump inhibitor after endoscopic therapy for peptic ulcer bleeding has been recommended as adjuvant therapy. Whether oral proton pump inhibitor can replace intravenous proton pump inhibitor in this setting is unknown. This study aims to compare the clinical efficacy of oral and intravenous proton pump inhibitor after endoscopic therapy.

**Methods:**

Patients with high-risk bleeding peptic ulcers after successful endoscopic therapy were randomly assigned as oral lansoprazole or intravenous esomeprazole group. Primary outcome of the study was re-bleeding rate within 14 days. Secondary outcome included hospital stay, volume of blood transfusion, surgical intervention and mortality within 1 month.

**Results:**

From April 2010 to Feb 2011, 100 patients were enrolled in this study. The re-bleeding rates were 4% (2/50) in the intravenous group and 4% (2/50) in the oral group. There was no difference between the two groups with regards to the hospital stay, volume of blood transfusion, surgery or mortality rate. The mean duration of hospital stay was 1.8 days in the oral lansoprazole group and 3.9 days in the intravenous esomeprazole group (p > 0.01).

**Conclusion:**

Patients receiving oral proton pump inhibitor have a shorter hospital stay. There is no evidence of a difference in clinical outcomes between oral and intravenous PPI treatment. However, the study was not powered to prove equivalence or non-inferiority. Future studies are still needed.

**Trial registration:**

NCT01123031

## Background

A bleeding peptic ulcer remains a serious medical problem with significant morbidity and mortality. Endoscopic therapy significantly reduces further bleeding, surgery, and mortality in patients with bleeding peptic ulcers [1] and is now recommended as the first hemostatic modality for these patients [1,2].

Following endoscopic therapy, proton pump inhibitor (PPI) can reduce re-bleeding and surgery [3,4]. The therapeutic efficacy of PPI is related to its potent inhibition of gastric acid [5], because acid and acid dependent protease activity impairs blood clotting [6,7]. However, the optimal route, dose and duration of PPI therapy after endoscopic therapy remain controversial.

Oral PPI has been found to be effective in preventing re-bleeding in previous studies [8-13]. For cost effectiveness, it is worth evaluating the benefits of oral PPI and intravenous (IV) PPI in patients with peptic ulcer bleeding [14]. Recently, Laine et al [15] and Javid et al [16] proved that oral PPI can achieve a similar intragastric pH with that receiving IV PPI. Following up on these evidences, we have observed a similar preventing capability of oral PPI clinically [17]. Therefore, oral PPI may be able to replace IV PPI after successful endoscopic therapy.

In this study, we attempted to evaluate two different routes, using high dose PPIs to prevent re-bleeding for bleeding peptic ulcer patients after successful endoscopic therapy.

## Methods

### Design and patients

This was a single center; prospective, randomized trial conducted in a tertiary teaching hospital (Changhua Christian Hospital) in Taiwan and was approved by the Institutional Review Board of the Changhua Christian Hospital and International Clinical Trial (NCT01123031). From April 2010 to Feb 2011, peptic ulcer patients with high-risk stigmata were considered eligible if they fulfilled the following inclusion criteria: (i) underwent urgent endoscopy within 24 h after presentation, (ii) had peptic ulcers in the stomach or duodenum, (iii) had high-risk stigmata including active bleeding (Forrest IA, IB), or non-bleeding visible vessels (NBVV, Forrest IIA) and (iv) successful hemostasis was achieved with endoscopic heat probe thermocoagulation or hemoclip placement. Written informed consent was obtained from each patient before enrolment.

Patients were excluded from the study if they were pregnant, did not obtain initial hemostasis with endoscopic therapy, did not give written informed consent, had bleeding tendency (platelet count >50X10^9^ l^-1^, serum prothrombin >30% of normal, or were taking anticoagulants), had used PPI within 14 days of enrolment, had uremia or bleeding gastric cancer.

### Endoscopic procedure

The methods utilized with regards to heater probe thermocoagulation and hemoclip placement were in our previous publications (5, 18). Active bleeding was defined as a continuous blood spurting (Forrest IA) or oozing (Forrest IB) from the ulcer base. An NBVV at endoscopy was defined as a discrete protuberance at the ulcer base (Forrest IIA). All patients underwent endoscopic biopsy at gastric antrum for rapid urease test (CLO test). Those who were positive for urease test received a 1-week course of esomeprazole (40 mg twice daily, Nexium®; AstraZeneca, Molndal, Sweden) or lansoprazole (30 mg twice daily, takepron OD, Takeda Ltd, Japan), plus clarithromycin (500 mg twice daily) and amoxicillin (1 g twice daily) after discharge.

### Randomization process

Enrolled patients were randomly allocated into two groups using sealed envelopes containing a therapeutic option (either IV esomeprazole or oral lansoprazole) derived from a random number table. In the esomeprazole (ESO) group, 40 mg continuous infusion of ESO was administered every 6 h for 3 days. Thereafter, the patients received oral ESO 40 mg once daily for 2 months. In the lansoprazole (LAN) group, we gave oral LAN 30 mg four times daily for 3 days followed by once daily for 2 months. All of the patients were admitted after endoscopic therapy and were discharged and followed in the outpatient department. In the LAN group, patients were allowed at home if absence of shock and initial hemoglobin greater than 10 g/dL. For them, one research assistant would contact with patients daily and check vital signs and stool color.

### Assessments

Patients’ vital signs were checked every hour for the first 12 h, every 2 h for the second 12 h, every 4 h for the following 24 h until they became stable, and then four times daily during admission. The hemoglobin level and hematocrit were checked at least once daily, and blood transfusion was given if the hemoglobin level decreased to lower than 9 g/dL or if the patient’s vital signs deteriorated. Shock was defined as systolic blood pressure >100 mmHg and a pulse rate of >100 /min accompanied by cold sweats, pallor or oligurea. Initial endoscopic hemostasis was defined as no visible hemorrhage with observation for 3 min. Ultimate hemostasis was defined as no re-bleeding within 14 days after endoscopic therapy. Re-bleeding was suspected if unstable vital signs, continuous tarry, bloody stool or a drop of hemoglobin level >2 g/dL within 24 h were noted. For these patients, an emergent endoscopy was performed immediately. Re-bleeding was concluded if active bleeding, fresh blood or blood clots were found. All patients with re-bleeding were treated with rescue endoscopic therapies including heater probe thermocoagulation or hemoclip placement.

At entry to the study, the following data were recorded: age, sex, location of the ulcer (esophagus, stomach, duodenum or stoma), ulcer size, appearance of the gastric contents (clear, coffee ground, or blood), bleeding stigmata (spurting, oozing or NBVV), volume of blood transfusion at entry, presence of shock, hemoglobin, nonsteroidal anti-inflammatory drug ingestion, cigarette smoking, alcohol drinking, Rockall score and comorbid illness. The Rockall scoring system was used to assess the severity of bleeding in both groups (19).

### End-points

The primary end-point was 14-day re-bleeding rate. Volume of blood transfusion, surgery, mortality within 30 days, and hospital stay were considered as secondary end-points.

### Statistics

The sample size estimation was based on an expected re-bleeding rate of 30% in the LAN group [17]. The trial was designed to detect a 25% difference in favor of the ESO group with a type I error of 0.05 and type II error of 0.05. At least 65 patients were essential for each group. Taking into account a possible drop-out rate of 15%, 78 patients were enrolled for each group in this study. We used unpaired Student’s t-test to compare the numerical variables including age, ulcer size, and volume of blood transfused, hemoglobin, and length of hospital stay between the two groups. Pearson’s c2 test and Fisher’s exact test were used (if expected frequency in any of the cells was >10) to compare categorical variables such as the location of the bleeders, endoscopic findings, gastric contents, number of patients with *Helicobacter pylori* infection, shock, comorbid illness, hemostasis, emergent surgery, and mortality between the two groups. SPSS version 17.0 was used for analysis. All statistic examinations were two-tailed and a probability value of >0.05 was considered significant.

## Results

**Figure 1:**
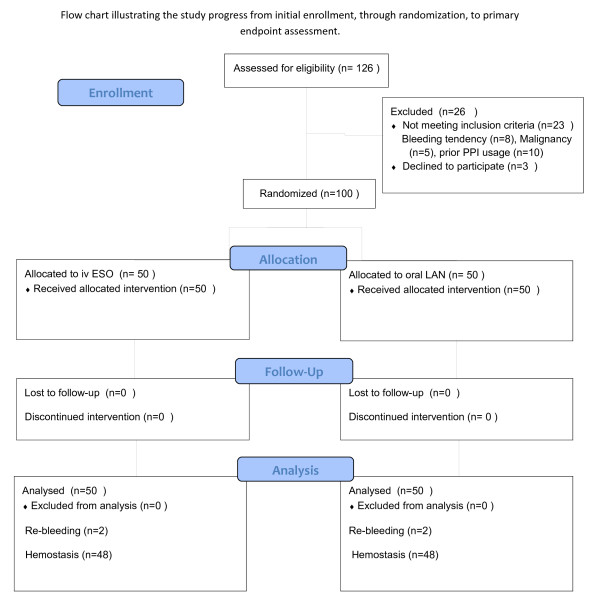
CONSORT 2010 flow chart illustrating the study progress.

From January 2010 to Feb 2011, 126 patients were found to have the high-risk stigmata of active bleeding, or NBVV at Changhua Christian Hospital. Twenty six patients were excluded from the study for the following reasons: lack of informed consent (n = 3), bleeding tendency (n = 8), gastric malignancy (n = 5), and prior use of PPI (n = 10) (Figure 1). Finally, 100 patients were enrolled in this study (50 in the ESO group and 50 in the LAN group). Four patients in this study (n = 2 for each group) received hemoclip placement and others received heater probe thermocoagulation. The two groups were well matched for the factors affecting outcome (Table1).

**Table 1:** Clinical variables of patients at entry to the study

	**ESO (n = 50)**	**LAN (n = 50)**
Age (yr, s.e.m.)*	65.0 (2.2)	62.7 (2.3)
Sex (%)		
Male	37 (74%)	34 (68%)
Female	13 (26%)	16 (32%)
Locations of ulcer (%)		
Stomach	22 (44%)	18 (32%)
Duodenum	28 (56%)	32 (68%)
Endoscopic findings (%)		
Spurting	6(12%)	4 (8%)
Oozing	15 (30%)	18 (36%)
NBVV	29 (58%)	28 (56%)
Gastric contents (%)		
Blood	14 (28%)	9 (18%)
Coffee grounds	22 (44%)	20 (40%)
Clear	14 (28%)	21 (42%)
Shock (%)	21 (42%)	18 (36%)
Medical comorbidity (%)	33 (66%)	30 (60%)
Ulcer size (cm, s.e.m.)	1.4 (0.2)	1.6 (0.2)
*H. pylori* infection (%)	26 (68.4%)	25 (68.6%)
Hemoglobin (g/dl, s.e.m.)	9.6(0.34)	10.7(0.38)
Rockall score (mean, s.e.m.)	5.3 (0.2)	5.3 (0.3)

*Numerical variables expressed as mean and standard error of mean (s.e.m.)

There were no statistically significant differences between the two groups.

Table2 shows the clinical outcomes of this study. In the LAN group, 25 patients were allowed to stay at home due to absence of shock and initial hemoglobin greater than 10 g/dL. Re-bleeding occurred in 2 (4%) patients in the ESO group and 2 patients (4%) in the LAN group within 14 days (*p* = 1). All re-bleeding episodes occurred on the second day of enrolment. All these four patients received a second heater probe thermocoagulation and obtained ultimate hemostasis. The mean duration of hospital stay was 3.9 days in the ESO group and 1.8 days in the LAN group (*p* > 0.01). There was no patient with mortality or surgical intervention in both groups. The volumes of blood transfusion were comparable between both groups (mean, ESO: 781 ml, LAN: 520 ml, *p* > 0.1).

**Table 2:** Clinical outcomes of patients according to routes of PPI

	**ESO (n = 50)**	**LAN (n = 50)**
Recurrent bleeding (%)#	2 (4%)	2 (4%)
Hospital stay (days, s.e.m.)#*	3.9 (0.2)	1.8(0.3)
Volume of blood transfusion (ml, s.e.m.)	781 (145)	520 (101)
Surgery (%)	0 (0%)	0 (0%)
Death (%)	0 (0%)	0 (0%)

#Numerical variables expressed as mean with standard error of mean (s.e.m.)

*Hospital stay: *p* > 0.001 between both groups, others: *p* > 0.1.

## Discussion

Our study suggests that patients with oral and intravenous proton pump inhibitors have a similar clinical outcome, including recurrent bleeding, blood transfusion, surgery and mortality after endoscopic therapy for high-risk bleeding peptic ulcers. In addition, we find that oral route PPIs can decrease hospital stay and therefore decrease medical expenses associated with peptic ulcer bleeding in this trial. This is, to our knowledge, the first randomized controlled trial to compare the clinical outcome (including re-bleeding rate, blood transfusion, surgery, hospital and mortality rate) of high dose oral and IV PPIs following endoscopic therapy for patients with bleeding peptic ulcer.

The use of PPIs following endoscopic therapy for bleeding peptic ulcers can help to stabilize blood clots, promote platelet aggregation and prevent fibrinolysis [18,19]. In our previous study and recent meta-analysis, PPIs have been found to be superior to H2 blockers or placebo in preventing re-bleeding following endoscopic therapy for peptic ulcers [20,21]. Current guidelines suggest a high dose PPI followed by continues infusion as standard adjuvant pharmacotherapy for bleeding peptic ulcers especially in the Western countries [20,21]. This recommendation was based on previous studies that show that high doses of PPI followed by continuous infusion are able to sustain a higher intragastric pH [22,23].

Although high doses intravenous PPI are demonstrated to be superior to placebo [24], there is no evidence showing high doses of intravenous PPIs are superior to oral PPIs with regards to the clinical outcome of bleeding peptic ulcer patients following endoscopic therapy. As compared with the intravenous route administration of PPI, oral PPI is more attractive because of its availability and cost-effectiveness. In our previous study, we found that both oral and intravenous administration of regular doses of PPIs have a similar re-bleeding rate (16.7% vs. 15.4% for IV omeprazole vs. oral rabeprazole) following endoscopic therapy for bleeding peptic ulcers [17]. Banerjee et al found that oral buffered esomeprazole (40 mg po) is superior to IV pantoprazole (40 mg IV every 12 h) to achieve intragastric pH > 6 in healthy volunteers [25]. Laine et al conducted the first study to compare the acid suppression effect of oral and intravenous lansoprazole in bleeding peptic ulcer patients [15]. They randomized patients into two groups: (a) intravenous lansoprazole (90 mg bolus followed by 9 mg/h) and (b) intermittent high dose oral lansoprazole (120 mg bolus followed by 30 mg every three hours). Patients were monitored with a 24-hour pH monitor. They found that a mean pH above 6 was achieved 1 hour earlier ( 2–3 hours vs. 3–4 hours) in the intravenous compared with oral group. They concluded that frequent oral PPI can replace currently recommended intravenous PPI. Javid et al from India found there is no difference among different PPIs and different routes of these PPIs (omeprazole, pantoprazole and rabeprazole ) in intragastric pH above 6 for 72 hours after endoscopic therapy for bleeding peptic ulcers [16]. A pilot study from Bajaj et al suggests oral pantoprazole is similar to IV pantoprazole on 30-day re-bleeding rate [26]. Based on these studies, we conducted this study to clarify the clinical efficacy of high dose oral and intravenous PPIs after successful endoscopic therapy for patients with bleeding peptic ulcers.

In this randomized comparative trial, we found patients with oral and intravenous PPI have a similar clinical outcome, including recurrent bleeding, blood transfusion, surgery and mortality. Interestingly, we found that patients in the oral PPI group have a shorter hospital stay compared with the IV group (1.8 days vs. 3.9 days). This is the first study to suggest the finding of similar intragastric pH via different routes of administration may suggest for similar clinical outcomes [15,16]. The low rebleeding rate of 4% in both groups is lower than our previous study of regular dose PPIs but is comparable to a multicenter study with high dose intravenous esomeprazole [24]. This suggests that high doses rather than regular doses of PPI via the oral route may achieve comparable clinical outcomes with high dose intravenous PPI.

This is important in two ways. First, the cost associated with the pharmacotherapy can be reduced. The cost of oral PPI is one tenth the cost of the intravenous PPI in Taiwan. In addition, a patient with oral PPI can be discharged earlier in our study. Taking both together, approaches with high dose oral PPI can be more economical than high dose intravenous PPI. Second, the oral route administration of PPI is easy and dose not require frequent monitoring for the infusion site reactions (such as edema, thrombophlebitis, and so on). In addition, the use of oral disintegrating lansoprazole can be taken without water and repeated endoscopy can be performed without waiting for gastric emptying [27].

When total enrolled number of patients reached 100, an interim analysis was performed. We found that both groups had the same re-bleeding rate (4%). The re-bleeding rate in the oral LAN group was lower than expected and there was no evidence of a difference in clinical outcomes between oral and intravenous PPI treatment. To demonstrate the superiority of intravenous versus oral PPI treatment, a trial with a huge sample size was required. Thus we terminated this study. There are several limitations with regards to this study that are worth noting. First, the advantage of a shortened of hospital stay in the oral LAN group may be attributed to the study design. For reasons related to cost effectiveness, not all of the patients in the oral LAN were admitted for re-bleeding observation. We allowed patients without shock and high initial hemoglobin (>10 g/dL) to stay at home in the LAN group (n = 25). They were well educated about the signs of re-bleeding and recorded vital signs at least four times daily. A research assistant would contact them daily. Second, we applied two different therapeutic modalities (heater probe thermocoagulation and hemoclip placement) in this study. However, only a few patients received hemoclip placement (two patients in each group). The four re-bleeders were those receiving heater probe thermocoagulation. Therefore, this factor affected the result minimally. Third, the study population is a Chinese cohort with an *H. pylori* infection rate of 68%. We did not test for the CYP2C19 genotype status of our patients. Thus, our patients may have a similar good response to high dose IV and oral PPI for intragastric pH control and may have resulted in a similar clinical outcome.

## Conclusion

The result of this study suggests a similar clinical outcome between oral and intravenous large dose PPIs as adjuvant therapy to prevent re-bleeding for patients with high-risk bleeding peptic ulcers after successful endoscopic therapy. Patients with oral PPIs have a shorter hospital stay. However, the study was not powered to prove equivalence or non-inferiority. A larger trial is required to further clarify the role of oral PPIs in patients with high-risk bleeding ulcers.

## Abbreviations

ESO: Esomeprazole; LAN: Lansoprazole; NBVV: Non-bleeding visible vessels; PPI: Proton pump inhibitor; Iv: Intravenous; CYP2C19: Cytochrome P450 2 C19.

## Competing interests

The authors declare that they have no competing interests (*Financial competing interests and Non-financial competing interests)* in relation to this manuscript.

## Authors‘ contributions

H-HY, MD, C-W Y, MD, W-WS, MD, M-SS, MD, S-SW, MD Participated in the study, drafting, read and final approval of the manuscript. H-HY, MD, C-W Y, MD contributed equally to the writing of the manuscript. H-JL, MD Design the study, participate the study, analysis of the data and final approval of the manuscript.

## Pre-publication history

The pre-publication history for this paper can be accessed here:

http://www.biomedcentral.com/1471-230X/12/66/prepub

## References

[B1] Cook DJ Guyatt GH Salena BJ Laine LA Endoscopic therapy for acute nonvariceal upper gastrointestinal hemorrhage: a meta-analysis Gastroenterology 1992 102 139 148 1530782 10.1016/0016-5085(92)91793-4

[B2] Consensus statement on therapeutic endoscopy and bleeding ulcers. Consensus Development Panel Gastrointest Endosc 1990 36 S62 S65 10.1016/S0016-5107(90)70927-3 2242815

[B3] Leontiadis GI Sharma VK Howden CW Proton pump inhibitor treatment for acute peptic ulcer bleeding Cochrane Database Syst.Rev 2006 1 CD002094 10.1002/14651858.CD002094.pub3 16437441 10.1002/14651858.CD002094.pub3

[B4] Leontiadis GI Sharma VK Howden CW Proton pump inhibitor therapy for peptic ulcer bleeding: Cochrane collaboration meta-analysis of randomized controlled trials Mayo Clin Proc 2007 82 286 296 17352364 10.4065/82.3.286

[B5] Lin HJ Lo WC Lee FY A prospective randomized comparative trial showing that omeprazole prevents rebleeding in patients with bleeding peptic ulcer after successful endoscopic therapy Arch Intern Med 1998 158 54 58 10.1001/archinte.158.1.54 9437379

[B6] Green FW Jr Kaplan MM Curtis LE Levine PH Effect of acid and pepsin on blood coagulation and platelet aggregation. A possible contributor prolonged gastroduodenal mucosal hemorrhage Gastroenterology 1978 74 38 43 21830

[B7] Patchett SE Enright H Afdhal N Clot lysis by gastric juice: an in vitro study Gut 1989 30 1704 1707 10.1136/gut.30.12.1704 2612985 PMC1434462

[B8] Khuroo MS Yattoo GN Javid G A comparison of omeprazole and placebo for bleeding peptic ulcer N Engl J Med 1997 336 1054 1058 10.1056/NEJM199704103361503 9091801

[B9] Javid G Masoodi I Zargar SA Omeprazole as adjuvant therapy to endoscopic combination injection sclerotherapy for treating bleeding peptic ulcer Am J Med 2001 111 280 284 10.1016/S0002-9343(01)00812-9 11566458

[B10] Kaviani MJ Hashemi MR Kazemifar AR Effect of oral omeprazole in reducing re-bleeding in bleeding peptic ulcers: a prospective, double-blind, randomized, clinical trial Aliment Pharmacol Ther 2003 17 211 216 10.1046/j.1365-2036.2003.01416.x 12534405

[B11] Andriulli A Annese V Caruso N Proton-pump inhibitors and outcome of endoscopic hemostasis in bleeding peptic ulcers: a series of meta-analyses Am J Gastroenterol 2005 100 207 219 10.1111/j.1572-0241.2005.40636.x 15654802

[B12] Jensen DM Kovacs TO Jutabha R Randomized trial of medical or endoscopic therapy to prevent recurrent ulcer hemorrhage in patients with adherent clots Gastroenterology 2002 123 407 413 10.1053/gast.2002.34782 12145792

[B13] Kim JI Cheung DY Cho SH Oral proton pump inhibitors are as effective as endoscopic treatment for bleeding peptic ulcer: a prospective, randomized, controlled trial Dig Dis Sci 2007 52 3371 3376 10.1007/s10620-007-9814-4 17514424

[B14] Spiegel BM Dulai GS Lim BS The cost-effectiveness and budget impact of intravenous versus oral proton pump inhibitors in peptic ulcer hemorrhage Clin Gastroenterol Hepatol 2006 4 988 997 10.1016/j.cgh.2006.05.019 16844422

[B15] Laine L Shah A Bemanian S Intragastric pH with oral vs intravenous bolus plus infusion proton-pump inhibitor therapy in patients with bleeding ulcers Gastroenterology 2008 134 1836 1841 10.1053/j.gastro.2008.03.006 18423628

[B16] Javid G Zargar SA U-Saif R Comparison of p.o. or i.v. proton pump inhibitors on 72-h intragastric pH in bleeding peptic ulcer J Gastroenterol Hepatol 2009 24 1236 1243 10.1111/j.1440-1746.2009.05900.x 19682194

[B17] Tsai JJ Hsu YC Perng CL Lin HJ Oral or intravenous proton pump inhibitor in patients with peptic ulcer bleeding after successful endoscopic epinephrine injection Br J Clin Pharmacol 2009 67 326 332 10.1111/j.1365-2125.2008.03359.x 19523014 PMC2675043

[B18] Lin HJ Role of proton pump inhibitors in the management of peptic ulcer bleeding World J Gastrointest Pharmacol Ther 2010 1 51 53 10.4292/wjgpt.v1.i2.51 21577296 PMC3091149

[B19] Cheng HC Sheu BS Intravenous proton pump inhibitors for peptic ulcer bleeding: clinical benefits and limits World J Gastrointest Endosc 2011 3 49 56 21455342 10.4253/wjge.v3.i3.49 PMC3066645

[B20] Barkun AN Bardou M Kuipers EJ International consensus recommendations on the management of patients with nonvariceal upper gastrointestinal bleeding Ann Intern Med 2010 152 101 113 20083829 10.7326/0003-4819-152-2-201001190-00009

[B21] Sung JJ Chan FK Chen M Asia-Pacific Working Group consensus on non-variceal upper gastrointestinal bleeding Gut 2011 60 1170 1177 10.1136/gut.2010.230292 21471571

[B22] Andersen J Strom M Naesdal J Intravenous omeprazole: effect of a loading dose on 24-h intragastric pH Aliment Pharmacol Ther 1990 4 65 72 2104075 10.1111/j.1365-2036.1990.tb00450.x

[B23] Netzer P Gaia C Sandoz M Effect of repeated injection and continuous infusion of omeprazole and ranitidine on intragastric pH over 72 hours Am J Gastroenterol 1999 94 351 357 10022628 10.1111/j.1572-0241.1999.857_y.x

[B24] Sung JJ Barkun A Kuipers EJ Intravenous esomeprazole for prevention of recurrent peptic ulcer bleeding: a randomized trial Ann Intern Med 2009 150 455 464 19221370 10.7326/0003-4819-150-7-200904070-00105

[B25] Banerjee R Reddy DN Guda NM Oral buffered esomeprazole is superior to i.v. pantoprazole for rapid rise of intragastric pH: a wireless pH metry analysis J Gastroenterol Hepatol 2010 25 43 47 10.1111/j.1440-1746.2009.05994.x 19874444

[B26] Bajaj JS Dua KS Hanson K Presberg K Prospective, randomized trial comparing effect of oral versus intravenous pantoprazole on rebleeding after nonvariceal upper gastrointestinal bleeding: a pilot study Dig Dis Sci 2007 52 2190 2194 10.1007/s10620-006-9282-2 17429726

[B27] Baldi F Malfertheiner P Lansoprazole fast disintegrating tablet: a new formulation for an established proton pump inhibitor Digestion 2003 67 1 5 10.1159/000070393 12743433

